# Recent Advances in Bromine Complexing Agents for Zinc–Bromine Redox Flow Batteries

**DOI:** 10.3390/ma16237482

**Published:** 2023-12-02

**Authors:** Uxua Jiménez-Blasco, José Carlos Arrebola, Alvaro Caballero

**Affiliations:** 1 Departamento de Química Inorgánica e Ingeniería Química, Instituto Químico para la Energía y el Medioambiente (IQUEMA), Universidad de Córdoba, 14014 Córdoba, Spain; uxua.jimenez@unavarra.es (U.J.-B.); alvaro.caballero@uco.es (A.C.); 2 Departamento de Ciencias, Universidad Pública de Navarra, Campus de Arrosadía, 31006 Pamplona, Spain; 3 Institute for Advanced Materials and Mathematics (INAMAT2), Universidad Pública de Navarra, Campus de Arrosadía, 31006 Pamplona, Spain; 4 Departamento de Didácticas Específicas, Universidad de Córdoba, 14014 Córdoba, Spain

**Keywords:** redox flow battery, zinc–bromine flow battery, bromine complexing agent

## Abstract

The development of energy storage systems (ESS) has become an important area of research due to the need to replace the use of fossil fuels with clean energy. Redox flow batteries (RFBs) provide interesting features, such as the ability to separate the power and battery capacity. This is because the electrolyte tank is located outside the electrochemical cell. Consequently, it is possible to design each battery according to different needs. In this context, zinc–bromine flow batteries (ZBFBs) have shown suitable properties such as raw material availability and low battery cost. To avoid the corrosion and toxicity caused by the free bromine (Br_2_) generated during the charging process, it is necessary to use bromine complexing agents (BCAs) capable of creating complexes. As an overview, the different BCAs used have been listed to compare their behavior when used in electrolytes in ZBFBs. In addition, the coulombic and energy efficiencies obtained have been compared.

## 1. Introduction

Renewable energy sources are very important to reduce greenhouse gas emissions and to fight against climate change. As a complement to renewable energy sources, energy storage systems that can be used to regulate power generation are necessary to balance and guarantee the continuity of operation, since renewable energies are intermittent sources [1,2]. Regarding these energy storage systems, during off-peak hours, when the demand is lower than generation, energy is stored, and, at peak times, when the demand is higher than generation, the remaining load is supplied by the energy storage system [3,4,5].

Among energy storage systems, there are different technologies [4,6], such as mechanic, thermal, magnetic or electrochemical. The latter transform electrical energy into chemistry via redox chemical reactions. There three types of these systems: batteries (including non-rechargeable primary and rechargeable, or secondary batteries), electrochemical capacitors (electric double layer capacitors and pseudo-capacitors) and fuel cells [7,8,9].

As for batteries, they are used both as a storage system and as an energy transformation system. This type of electrical storage system features a high specific energy and a constant output voltage. There are different technologies within secondary batteries, depending on the redox pair. The best known and most commercialized are those based on Pb, Li, Ni and Na, but, in recent years, flow batteries, more specifically those based on Zn-Br (ZBFBs), have gained great importance. Batteries based on Li have a higher energy density, energy efficiency and power density compared to the rest of the technologies, followed by those based on Na, Pb and Ni. In the case of ZBFBs, their values are above Ni and Pb batteries, although the most interesting thing about this technology, in addition to the energy values, is the low cost of the materials and their abundance and availability. In addition to this, because the electrolyte tanks are located outside the electrochemical stack, they have high scalability. As mentioned above, one of the advantages of ZBFBs is their use of abundant and available materials, which makes their price per kW/h lower compared to other technologies, and the cost is estimated between 350 to 600 dollars [10]. The environmental impact associated with the assembly of ZBFBs has been studied by He et al. [11], and has been seen to be less compared to other technologies [12,13,14,15].

Redox flow batteries (RFBs) are rechargeable devices which are used for energy storage and the electrolytes are pumped through the electrochemical cell to transform the chemical energy into electric energy. A typical RFB usually has two electrolyte tanks for energy storage, a cell or several cells (stack) for the electrochemical reaction and pumps for the flow of the electrolyte between the tanks and the stack. The difference between RFB technology and other battery systems is that in RFBs, the energy is stored in electrolyte solutions, and the system capacity is determined by the redox pair concentration and the volume of the electrolytes. In addition, the system’s power is determined by the electrode’s surface. Consequently, the power and the capacity are separated and offer flexibility to build battery systems under different working conditions [16,17,18]. If RFB technology is compared with other technologies, RFBs have a lower power and current density that is not appropriate for traction and mobile applications. Usually, RFBs are used in stationary applications such as homes, smart grids, or telecommunications [17,18,19].

### 1.1. RFB Description

**Figure 1:**
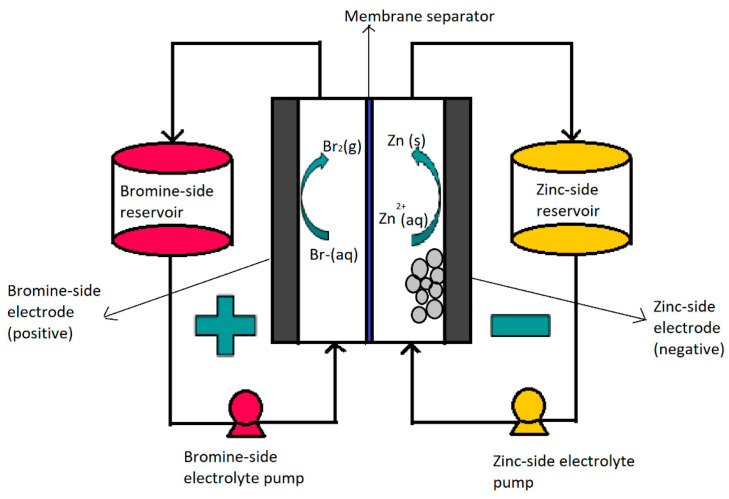
Operating diagram of a redox flow battery: ZBFB system. (Reproduced with permission from Ref. [21] under the Creative Commons CC BY license).

The transformation of chemical energy into electrical energy occurs by pumping the electrolyte from the reservoir to the stack, where the redox reactions occur. The anolyte (negative electrolyte) flows in the negative semi-cell, the catholyte (positive electrolyte) flows in the positive half-cell and the reduction–oxidation reactions occur at the electrode–electrolyte interface. In the oxidation reaction, electrons are generated and moved by the external circuit. For the reduction reaction to happen, the electrons are accepted into the other half-cell. A scheme can be seen in Figure 1 (example of a zinc–bromine system). For secondary batteries, the chemical reactions must be reversed during discharge. In this way, as one solution reduces, the other oxidizes, creating an electric current that passes through the externally installed electrical circuit. The potential difference between the redox pair of both half-cells determines the electromotive force or voltage of the cell. Both half-cells are separated by a membrane, to prevent the self-discharge of the battery. Across the membrane, electrolytes exchange ions to maintain electroneutrality and electrolyte balance [18,20].

### 1.2. RFB Classification

RFB classification can be carried out according to the structure of the cell or according to the type of redox couple used, as shown in Table 1. Considering the structure of the cell, four types of RFB have been developed [6]: true or redox flow batteries, type 1 hybrid redox flow batteries, type 2 hybrid redox flow batteries and flow batteries without membranes.

The first type is characterized by all the active materials being dissolved in the electrolyte [22,23,24], so that the capacity of the battery is totally independent of its power. One of the best-known examples is the vanadium flow battery (VFB).

Those of type 1 are characterized by there being one phase change in the active species in the chemical reaction, depositing material in the solid phase on one of the electrodes. Consequently, the surface of the electrode limits the storage capacity as well as the amount of electrolyte. A typical example is zinc–bromine flow batteries (ZBFBs), in which during the charging stage, solid zinc is deposited on the anode surface [22,25].

In type 2, both half-reactions involve phase changes in the charge or discharge phase. An example of this type is a soluble lead flow battery (SLFB), in which during charging, the Pb^2+^ ions pass into solid compounds that are deposited on the surface of both electrodes [22,25,26].

Finally, in flow batteries without membranes [27,28], two liquids are pumped through a channel, storing or releasing energy via electrochemical reactions. Both solutions flow in parallel without mixing, without the need for a membrane. This technology is the least developed of all those cited.

**Table 1:** Main redox couples used in flow batteries [29].

Redox Couples	Redox Reaction	E^o^ (V)	Electrolyte
All-vanadium	anode: V^2+^ ←→ V^3+^ + e^−^ cathode: VO_2_^+^ + e^−^ ←→ VO^2+^	1.4	H_2_SO_4_/H_2_SO_4_
V/polyhalide	anode: V^2+^ ←→ V^3+^ + e^−^ cathode: ½Br_2_ + e^−^ ←→ Br^−^	1.3	VCl_3_-HCl/NaBr-HCl
Fe/Cr	anode: Fe^2+^ ←→ Fe^3+^ + e^−^ cathode: Cr^3+^ + e^−^ ←→ Cr^2+^	1.2	HCl/HCl
Br/polysulphure	anode: 2S_2_^2−^ ←→ S_4_^2−^ + e^−^ cathode: Br_2_ + 2e^−^ ←→ 2Br^−^	1.5	NaS_2_/NaBr
H_2_/Br_2_	anode: H_2_ ←→ 2H^+^ + e^−^ cathode: Br_2_ + 2e^−^ ←→ 2Br^−^	1.1	PEM^+^-HBr
Zn/Br	anode: Zn ←→ Zn^2+^ + 2e^−^ cathode: Br_2_ + 2e^−^ ←→ 2Br^−^	1.8	ZnBr_2_/ZnBr_2_
Zn/Ce	anode: Zn ←→ Zn^2+^ + 2e^−^ cathode: 2Ce^4+^ + 2e^−^ ←→ 2Ce^3+^	2.4	CH_3_SO_3_H/CH_3_SO_3_H

## 2. Zinc–Bromine Flow Batteries (ZBFBs)

A zinc–bromine flow battery (ZBFB) is a type 1 hybrid redox flow battery in which a large part of the energy is stored as metallic zinc, deposited on the anode. Therefore, the total energy storage capacity of this system depends on both the size of the battery (effective electrode area) and the size of the electrolyte storage tanks. For this reason, in this type of battery, the capacity and power are not totally decoupled [21,30].

During charge, the electrolyte is pumped into the cell, where bromine gas is formed in the cathode part due to the oxidation of the bromide, while in the anodic part, the zinc ions are reduced into metallic zinc and is deposited on the surface of the electrode [21,31,32].

Bromine has limited solubility in water, so a bromine complexing agent (BCA) or quaternary amine is added to the electrolyte to capture the bromine formed and prevent its evaporation (the boiling point of bromine is 58.8 °C). As the bromine–BCA complex forms, a denser and more viscous organic liquid phase immiscible with aqueous electrolyte is formed, which sinks to the bottom of the positive electrolyte (catholyte) tank [33].

In discharge, the organic phase must be mixed with the rest of the catholyte to transport and release the bromine molecules on the surface of the positive electrode. During discharge, the metallic zinc that has formed at the anode is oxidized into Zn^2+^ ions, dissolving in the anolyte (negative electrolyte) and releasing two electrons that are transported by the external circuit. The electrons return to the cathode and reduce the bromine molecules into bromide ions, which are soluble in the catholyte. The chemical process used to generate the electric current increases the concentration of zinc and bromide ions in both electrolytes [34].

These are the reactions that occur during charge [21,30,32,35,36]:

Cathode: 2Br^−^ ←→ Br_2_ + 2e^−^  E^0^ = 1.06 V

Anode: Zn^2+^ + 2e^−^ ←→ Zn^0^  E^0^ = −0.76 V

Global reaction: 2Br^−^ + Zn^2+^ ←→ Br_2_ + Zn^0^  E^0^ = 1.86 V

As mentioned above, the energy efficiency of this battery is around 70%, and it also offers one of the highest voltages of the redox pairs used in flow batteries (1.8 V), releasing two electrons per atom of zinc. In having such a high energy density, the weight and cost are reduced for the same storage capacity as a similar battery. However, since bromine is a highly toxic compound to inhale or absorb, it is necessary for a complete bromine capture process to take place within the cell. This is essential for the safety and efficiency of the system [37].

### ZFBF Components

ZBFBs consist of three main components: the electrochemical stack, the hydraulic circuit and the battery control system (BMS). The electrochemical cell is built of two half-cells, each of which is made up of electrodes in which the active materials are charged and discharged and supported by a suitable fluid dynamic design, through which the electrolyte flows. Both half-cells are separated by a membrane or separator [38]. Stacks are made up of several cells placed in parallel one on top of the other and electrically connected in series.

As for the electrodes, in ZBFBs, they are mostly composed of carbon-based materials such as graphite, carbon fibers or carbon nanotubes. The choice of appropriate materials is really important to obtain the desired behaviors. In this regard, several properties are essential, such as the conductivity or the surface characteristics to correctly deposit the metallic zinc during charging. On the other hand, the use of composites made up of carbonic-polymeric materials for the electrodes or the doping of graphene materials using heteroatoms is common to increase efficiency [30,36,39].

Another main component is the membrane. A selective porous membrane pulls apart both half-cells and allows the crossing of ions without allowing the species to mix, avoiding self-discharge. There are several types of membranes, in the form of fabrics, microporous paper, polymer, etc. [37,40].

As for the electrolyte, it must be remarked that it is the most essential component of the ZBFB, due to the fact that it contains the active species, responsible for the redox reaction and therefore for the difference in voltage or power generated. To avoid net species transfer, both electrolytes (positive or catholyte and negative or anolyte) have the same composition. Zinc bromide is the main component of the electrolyte. Its ions participate in the redox reaction that occurs on the surface of the electrode. ZnBr_2_ is usually used in concentrations between 1 and 3 M, while the pH is generally kept between 1 and 3.5 to avoid dendritic zinc deposition [41,42]. Furthermore, it is common to use other additional salts as support and to increase the conductivity of the electrolyte, which causes the pH to vary until reaching pH 4. Usually, salts with chlorides are used, due to the changing lability of the protons in the solution and charge transfer impedances at the anode surface [32,39]. Furthermore, the use of a complexing agent is essential to trap the free bromine (Br_2_) formed. Its importance lies in the fact that free bromine causes corrosion and toxicity, both among the main problems in ZBFBs. These complexing agent materials are extensively addressed shortly.

Finally, a characteristic element of this system is the hydraulic circuit, and consists of the electrolyte storage tanks (positive and negative) and pumps to pump the electrolyte flow through the electrochemical cell. The tanks are connected to the electrochemical cell via a series of valves and conduits. The electrolytes flow into the circuit, which contains the active species [16,43,44]. To conclude, it is essential to have electronic management over all electronic and hydraulic parameters (power control, valve opening, flow control and loading–uploading protocol), as carried out by the BMS, which allows this control in each of the cells that make up the stack, as well as in the full flow battery [45].

## 3. Bromine Complexing Agents (BCAs)

The use of quaternary amines is essential to act as bromine complexing agents (BCAs). The elemental bromine complexed with the quaternary amine is stored safely, forming an oily phase that remains immiscible with the rest of the aqueous electrolyte. The concentration molar ratio between the complexing agent and the ZnBr_2_ active material generally used is approximately 1:3; therefore, a 3 M-concentration electrolyte solution in ZnBr_2_ should contain approximately 1 M concentration of the BCA [21,42,46].

The use of a suitable BCA is very important when designing a battery with this technology, as it is a fundamental part of its proper functioning. One of the most important requirements is the fast kinetics of the complexation mechanism the between salt and Br_2_, avoiding the escape of the Br_2_ formed on the surface of the electrode from the cell, or even migration to the anode, thus causing the self-discharge of the ZBFB [17].

The Br_2_ formed during the charge process is complexed with the quaternary amine (QBr) in the polybromide form. Monobromide ions have been found to react with aqueous bromine, which develops during charging to form tribromide and polybromide ions, as shown by reactions [33,38,42]:

Br^−^ + Br_2_ → Br_3_^−^

Br_3_^−^ + Br_2_ → Br_5_^−^

Br_5_^−^ + Br_2_ → Br_7_^−^

Diffusion of bromine via the separator is not completely blocked and causes the primary self-discharge mechanism in a zinc half-cell. For this reason, the BCA must also be added to the negative electrolyte, as well as the positive electrolyte. This could have consequences on the deposition and growth of zinc at the anode. In addition to the kinetics of the Br_2_–QBr complexation mechanism, it is very important that the organic phase formed is totally immiscible with the aqueous electrolyte, because during the charge process, it is necessary for this phase to be deposited at the bottom of the storage tank [42]. For all these reasons, it is necessary to study complexing agents capable of improving the properties of those currently used.

Compounds known as ionic liquids (IL) have very interesting characteristics, which makes them ideal for use as BCAs in ZBFBs [42]. These compounds are low-temperature molten salts consisting of a large organic cation and inorganic or organic anion with a melting point less than 100 °C [47]. They are characterized by nonvolatility, nonflammability, a wide liquid range [48] and excellent chemical and thermal stability [49]. The unique characteristics of ILs make them proper alternatives to classical organic solvents and they have been tested in different areas like electrochemistry, liquid phase extractions, catalysis for clean technology and polymerization processes [48,50,51,52,53].

The general cation structures of ionic liquids are pyrrolidinium, sulfonium, ammonium, phosphonium, imidazolium and pyridinium, and the regular anions are halides, tetrafluoro-borate, bis(trifluoromethylsulfonyl)imide and hexafluoro phosphate [54,55].

In the case of ZBFBs, different ILs have been used as BCAs with successful results. In general, all these compounds are obtained via the Menshutkin reaction. This reaction is a special bimolecular nucleophilic substitution (SN2) or an N-alkylation where the exchange of substituents with different charges takes place on the sp3 carbon atom. In cases where the alkylation occurs between the tertiary amines and alkyl halides in the polar medium, this is known as the Menshutkin reaction (MR). According to the IUPAC, the Menshutkin reaction is defined as the trialkylammonium dehalogenation of alkyl halides [32,56,57,58,59,60,61].

### 3.1. Morpholinium-Based BCAs

This ammonium salt is one of the most studied and used as a bromine complexing agent in zinc–bromine flow batteries. This is because in the presence of this salt, the bromine transport phenomenon is lower compared to other salts [56,62,63].

The molecule consists of a six-membered ring in which one of them is an oxygen and the other a tetra-substituted nitrogen, which remains in the form of an ammonium ion.

#### 3.1.1. N-ethyl-N-methylmorpholinium Bromide (MEM-Br)

N-ethyl-N-methylmorpholinium bromide is an ammonium salt, with which methylmorpholinium is used as precursor. As mentioned above, this compound is obtained via the Menshutkin reaction and, besides methylmorpholinium, bromoethane is employed as an adduct and acetonitrile (ACN) as a solvent, with stirring for 48 h at 65 °C to obtain a yellow solid [64].

This ammonium salt is one of the most studied and used as a bromine complexing agent in zinc–bromine flow batteries because of the good performance of the ZBFB when it is used as a BCA [21,38]. Although MEM-Br is a commercial BCA, its synthesis process is not very simple or economical because it is necessary to conduct at a high temperature, with reflux and under a N_2_ atmosphere.

#### 3.1.2. N-methyl-N-propylmorpholinium Bromide (MPM-Br)

N-methyl-N-propylmorpholinium bromide is an analogous compound of MEM-Br because the only difference between the two BCAs is the length of the aliphatic chain. In the case of MPM-Br, there is an extra carbon, obtaining a propyl group instead of the ethyl group of MEM-Br [21].

As occurs with MEM-Br, MPM-Br is obtained via the Menshutkin reaction. In both cases, methylmorpholine functions as the base molecule, but for MPM-Br, 1-bromopropane works as the adduct and ACN as the solvent. 1-bromopropane must be added in excess to balance losses due to its evaporation. Table 2 shows the yields for the synthesis of MPM-Br under different conditions [21]. The different syntheses of this BCA are very interesting, since, in the case of MEM-Br, it does not require many steps to follow or a high temperature. In addition to this, one of the routes studied can be carried out at room temperature, which makes it ideal when scaling the process.

#### 3.1.3. 1-(2-Carboximethyl)-1-methylmorpholinium Bromide (CMMM-Br)

CMMM-Br is another IL with methylmorpholine as the base molecule, but in this case, 3-bromoprophanic acid is used as the adduct in the Menshutkin reaction. Therefore, one of the substituent groups is a carboxylic acid. To obtain this BCA, molar equivalents of the reagents must be mixed for 4 h. Next, the precipitate is purified by washing the salt with diethyl ether, filtered and dried in a vacuum at 60 °C [46]. This washing step in the synthesis process makes it more complicated than others.

### 3.2. Pirrolidinium-Based BCAs

Pyrrolidinium-based compounds are the other most studied ILs for use as BCAs in zinc–bromine flow batteries, due to their ability to form an effective complex with the free bromine generated during the battery-charging process. In this case, the molecule consists of a five-membered ring in which one is a tetra-substituted nitrogen, which remains in the form of an ammonium ion [65].

#### 3.2.1. 1-Ethyl-1-methylpyrrolidinium Bromide (MEP-Br)

Along with MEM-Br, this BCA is the most studied and used in commercial ZBFBs [66]. MEP-Br is obtained via a Menshutkin reaction using methylpyrrolidinium as the base molecule and bromoethane as the adduct in acetonitrile. N-methylpyrrolidine and bromoethane are dissolved in acetonitrile with stirring for 24 h and at room temperature [60,67]. Some of the reasons for the commercialization of this BCA are its simplicity, scalability and low cost compared to obtaining other products, although the biggest disadvantage of this BCA is its great hygroscopicity.

#### 3.2.2. 1-(2-Carboxymethyl)-1-methylpyrrolidinium Bromide (CMMP-Br)

CMMP-Br is an analog of MEP-Br because in this compound, one of the substituents is carboxylic acid. The Menshutkin reaction is carried out using methylmorpholinium as the base molecule and 3-bromopropanoic acid as the adduct, such as CMMM-Br. The conditions for this synthesis have been previously described, since they are the same as for obtaining CMMM-Br, but with the difference in the base molecule [46]. As with the synthesis of CMMM-Br, a washing step is necessary to obtain a purified product.

### 3.3. Imidazolium-Based BCAs

Imidazolium-based ILs are usually used in the electrolytes of other type of batteries such as lithium batteries [68], Al-S batteries [69] or fuel cells [70]. The molecule is an aromatic five-membered ring, where in positions 1 and 3, there is N instead C. Research is focused on the enhancement of the electrolyte of the batteries, specifically in obtaining a more effective bromine complexing agent, so it is common to test new ILs as BCAs [32].

#### 3.3.1. 1-Ethyl-3-methylimidazolium Bromide (EMI-Br)

EMI-Br is an imidazolium salt containing one substituent group on each of N. This compound has several applications, such as in drug formulation [71] and as an electrolyte in different batteries [72]. Due to its application as the electrolyte in batteries, its use in ZBFBs as a bromine complexing agent is of interest [64]. For the synthesis of EMI-Br, a Menshutkin reaction takes place, and 3-methylimidazole (base molecule) and bromoethane (adduct) must be added to a 2 L flask with stirring and reflux for 24 h at room temperature. After the exothermic reaction, the product is washed with diethyl ether (3 * 200 mL) and dried under a vacuum for 24 h. Subsequently, it is dissolved in 1.5 L water and decolorazed with charcoal (30 g, 70 °C and 24 h). Finally, the obtained product is lyophilized and dried under a vacuum (65 °C and 48 h) [34,73,74,75]. Therefore, this synthesis route is one of the ones with the most steps.

#### 3.3.2. 1,2-Dimethyl-ethylimidazolium Bromide (DMEI-Br)

DMEI-Br is an analogous compound to EMI-Br where the only difference between them is that there is an extra substituent group between the two atoms of nitrogen. Therefore, research has been carried out to study its behavior as a BCA [33]. DMEI-Br is synthesized via a Menshutkin reaction where 1,2-dimethylimidazole (base molecule) reacts with the bromoethane (adduct). First, 1,2-dimethylimidazole is dissolved and heated above 45 °C in a three-neck round-bottom flask fitted with a reflux condenser. Next, bromoethane is dropped into the heated 1,2-dimethylimidazole solution for over 12 h. The mixed solution is cooled back to room temperature and changed from a colorless solution into a white solid compound [76,77]. Although this BCA is similar to EMi-Br, it is easier to obtain.

#### 3.3.3. 1-(2-Hydroxyethyl)-3-methylimidazolium Bromine (C_2_OHMI-Br)

C_2_OHMI-Br is a novel BCA candidate and analogous compound to EMI-Br and DMEI-Br, with the difference in the substituent group. In this case, the Menshutkin reaction is carried out with 3-methylimidazole as the base molecule and bromoethanol as the adduct, with the corresponding purification and decolorization steps performed using the standard literature methods [34,75]. As with obtaining EMI-Br, washing, decoloring and drying steps are necessary.

### 3.4. Piperidinium-Based BCAs

Piperidinium is a six-membered ring molecule, of which one is a tetra-substituted nitrogen. As an ionic liquid (IL), it has interesting properties like non-volatility and a strong dissolving ability [78]. Recent studies have demonstrated the utility of piperidinium-based ILs for electrochemical systems due to their water immiscibility, high conductivity, thermal stability and electrochemical window [79].

#### 1-Ethyl-3-methylpiperidinium Bromide (C_2_MPip-Br)

As is mentioned above, piperidinium-based molecules show interesting properties for use in electrochemical systems. Therefore, C_2_MPip-Br could be a suitable candidate to use as a BCA in zinc–bromine flow batteries [74]. To obtain this novel BCA, a Menshutkin reaction is need with N-methylpiperidinium as the base molecule and bromoethane as the adduct. The mixture is refluxed at 75–80 °C for 48 h under nitrogen and cooled to room temperature. After this, the adduct is evaporated and the white solid washed with ethyl ether and recrystallized in fresh THF three times. Finally, it is dried under a vacuum for 48 h [75,80]. This is one of the most complex synthesis processes, since in addition to several steps to obtain a pure product, it is necessary to carry out the synthesis under a N_2_ atmosphere, like MEM-Br, and use the highest reaction temperature.

### 3.5. Pyridinium-Based BCAs

Pyridinium is an analog of benzene formed by a six-membered aromatic ring, in which there is a tetra-substituted nitrogen. As an ionic liquid, it has several applications as a corrosion inhibitor [81], as a solvent in organic chemistry [82,83] or in energy storage systems [84], among other things.

#### 3.5.1. 1-Ethylpyridinium Bromide (C_2_Py-Br)

C_2_Py-Br has been proposed as novel BCA because of its properties in creating complexes with free bromine. The molecule has an ethyl substituent group in the nitrogen, and the synthesis is carried out via a Menshutkin reaction using pyridine as the base molecule and bromoethane as the adduct, with the corresponding purification and decolorization steps performed using the standard literature methods [34,75]. Due to its low melting point, this BCA is in a liquid state and, like the rest of the BCAs studied, has an unpleasant fishy odor.

#### 3.5.2. 1-(2-Hydroxyethyl)-pyridinium Bromide (C_2_OHPy-Br)

As it is mentioned above, pyridinium-based compounds seem to be interesting compounds to use as BCAs in ZBFBs. C_2_OHPy-Br is an analogous compound of C_2_Py-Br with the only difference being that in this case, the substituent group is a hydroxyethyl group, and, in the Mensshutkin reaction, pyridine takes part as the base molecule and bromoethanol as the adduct [74,75], so the synthesis conditions are the same.

#### 3.5.3. 1-(Carboxymethyl)-pyridinium Bromide (CMMPy-Br)

CMMPy-Br has a carboxymethylic group as a substituent in the nitrogen and it is considered a potential BCA for ZBFBs. This compound is synthetized by refluxing one molar equivalent of pyridine with one molar equivalent of 2-bromoacetic acid in 40 mL of ethyl acetate for 4 h. The remaining solvent is removed using a desiccator, leaving the precipitate product [46]. Compared to the rest of the BCAs, the synthesis time of this compound is shorter.

### 3.6. Summary of All Salts Reported in This Review

Table 3 shows a summary of all the BCAs mentioned above, with their own synthesis conditions, including the precursors used, synthesis conditions in terms of temperature, time, the use of reflux or not, atmosphere used and use of vacuum for drying or not.

## 4. Study of Bromine Complexing Agents in ZBFBs

Zinc–bromine redox flow batteries (ZBFBs) should use a bromine complexing agent (BCA) as an additive for bromine stability, as shown below. However, the chemical and structural characteristics of the BCAs affect the overall performances of ZBFBs, such as the kinetic and reversibility of the redox couples, electrolyte conductivity, charge transfer resistance, zinc plating/stripping reversibility and bromine complexation capacity. Therefore, depending on the use of different BCAs, the coulombic and energy efficiency of the battery will change.

### 4.1. Bromine Complexation Capacity

BCAs combine with bromine molecules to form polybromide complex phases immiscible with the aqueous electrolyte using quaternary ammonium bromides [88]. As previously mentioned, the complexation mechanism is the following:

[Q·Br + Br_2_ ←→ Q·Br_3_] ←→ [Q·Br_3_ + Br_2_ ←→ Q·Br_5_] ←→…[Q·Br_2n−1_ + Br_2_ → Q(Br_2_)_n_·Br]

1 ≤ n ≤ 7

Raman spectra are used to analyze the polybromide formation and to compare the effectiveness of different BCAs. The polybromide-forming ability is evaluated comparing the polybromide growth from tribromide into pentabromide species. The measurement is carried out by extracting a sample of the charged catholyte. Then, the Raman spectra are measured at 532 nm, and Raman absorption for the polybromide anions observed at 160–310 cm^−1^ [33,89,90]. The Raman intensities for the polybromide species are shown in Table 4 [33].

**Figure 2:**
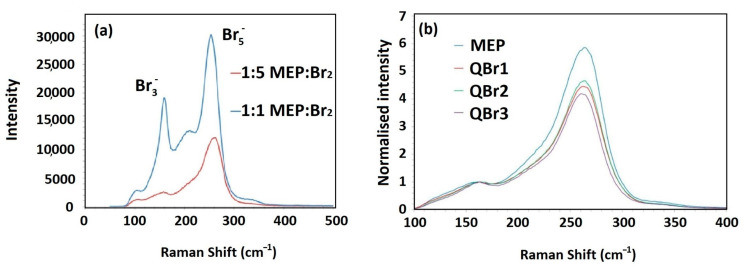
(**a**) Raman spectra of concentrated bromine solution with either 1:1 (blue) or 1:5 (red) molar equivalent of MEP-Br (T = 295 K) [46]; (**b**) Raman spectra normalized to Br_3_^−^ signals comparing Br_5_^−^ signal from immiscible phases formed with MEP, QBr1 (CMMPy-Br), QBr2 (CMMM-Br) and QBr3 (CMMP-Br) in 3:1 ratio of Br_2_: Q^+^Br_x_^−^ [46]. (Reproduced with permission from Ref. [46] under the Creative Commons CC BY license).

Figure 2a shows the Raman spectra from samples with a concentration ratio of 1:1 and 5:1 of Br_2_: MEP. Two peaks can be identified at 160 cm^−1^ and 250 cm^−1^ that coincide with the polybromide species Br_3_^−^ and Br_5_^−^, respectively. Thus, as the bromine content increments, the preferred polybromide species changes from Br_3_^−^ to Br_5_^−^ [46].

This method has been used to determine and compare the polybromide formation and bromine complexation capacity of several BCAs with commercial ones (MEM-Br and MEP-Br) and to without any BCA (pristine).

To compare the effectiveness of different BCAs with MEP-Br, the Br_3_^−^, peak is normalized to allow the proportion of the higher polybromide state of Br_5_^−^, to be emphasized. Figure 2b shows that with equal concentration ratios, MEP-Br still proves to be superior in forming the Br_5_^−^, species.

On the other hand, Y. Lee et al. [33] demonstrated that the bromine complexing ability of DMEI-Br for polybromide growth from tribromide into pentabromide species is distinctly greater compared to MEM-Br and MEP-Br. In addition, the Raman intensity ratio of pentabromide-to-tribromide resonances (I_5_/I_3_ at 259/183 cm^−1^) observed in the Raman spectra indicates the complexing ability in the preferential formation of the pentabromide over the tribromide species [33,91].

### 4.2. Influence on Zinc Electrodeposition

The bromine complexing agents in working ZBFB systems affect the zinc deposition and dissolution reactions. They can enhance the reaction kinetics of Zn^2+/^Zn redox species and improve the zinc-plating uniformity because of the electrostatic shield effect mechanism of the Q^+^ cations in the zinc half-cell [42,92]. Subsequently, a comparison of the effect of BCAs on the morphology of the zinc electrodeposited onto the electrode surface is very important.

**Figure 3:**
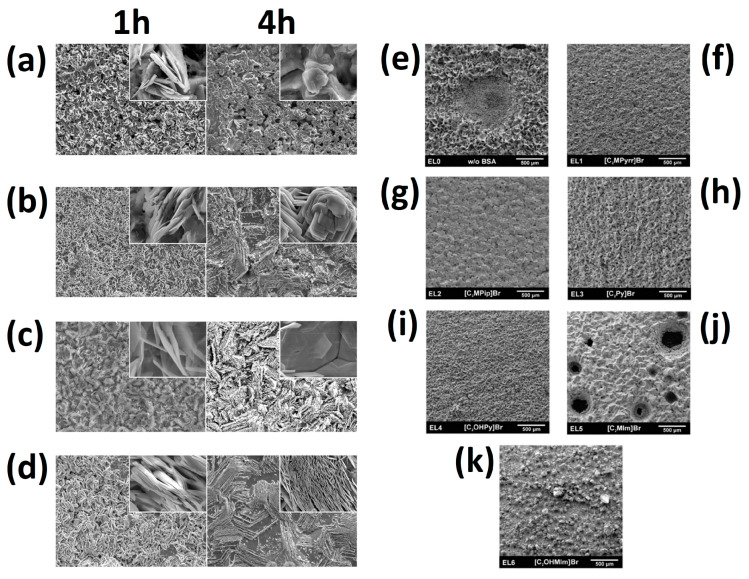
SEM images (×500/×10,000 magnification) of morphologies of electrodeposited zinc by charge time and BCA type: (**a**) pristine, (**b**) MEM-Br, (**c**) MEP-Br and (**d**) DMEI-Br [33]. SEM images of deposits acquired using various electrolytes: (**e**) EL0 without BCA, (**f**) EL1 MEP-Br, (**g**) EL2 C_2_MPip-Br, (**h**) EL3 C_c_MPy-Br, (**i**) EL4 C_2_OHPy-Br, (**j**) EL5 EMI-Br and (**k**) EL6 C_2_OHMI-Br [34]. ((**a**–**d**): reproduced with permission from Ref. [33]. © 2023 Elsevier B.V. All rights reserved. (**e**–**k**): reproduced with permission from Ref. [34]. © The Royal Society of Chemistry 2016).

Figure 3a–d show SEM images of the deposited zinc morphologies using different BCAs. In the case of MEM-Br, its morphology is transformed from a layered thin disc structure into a layered melting slurry-like form, while MEP-Br shows the morphology of a polygonal gravel texture into a rugged melting surface. For DMEI-Br, the zinc deposit is more compact and denser than that of MEP-Br and MEP-Br. A comparison of the morphologies indicates that the diffusion of DMEI-Br electrolyte is faster on the zinc metallic surface than MEM-Br and MEP-Br. This is because of the formation of stronger electrostatic attraction by the DMEI cation. Therefore, the structure of two heteroatom-containing imidazole rings of the DMEI-Br produces a stronger electrostatic shielding effect on charge, which allows the uniform electrodeposition of zinc and the effective suppression of the growth of zinc dendrite [33].

Figure 3e–k show the morphologies of the deposited zinc of other studied BCAs compared with the deposition of zinc without using BCA or using MEP-Br, C_2_MPip-Br, C_2_Py-Br, C_2_OHPr-Br, EMI-Br or C_2_MPy-Br, and shows the significant influence of the BCA on the deposition quality [28].

### 4.3. Electrochemical Properties: Cyclic Voltammetry (CV) Measurements and Electrochemical Impedance Spectroscopy (EIS)

CV and EIS experiments are carried out to check the effects of the BCAs on the electrochemical performances, such as the kinetic reaction and reversibility of the Zn^2+^/Zn and Br_2_/Br^−^ redox couples in a ZBFB electrolyte solution.

**Figure 4:**
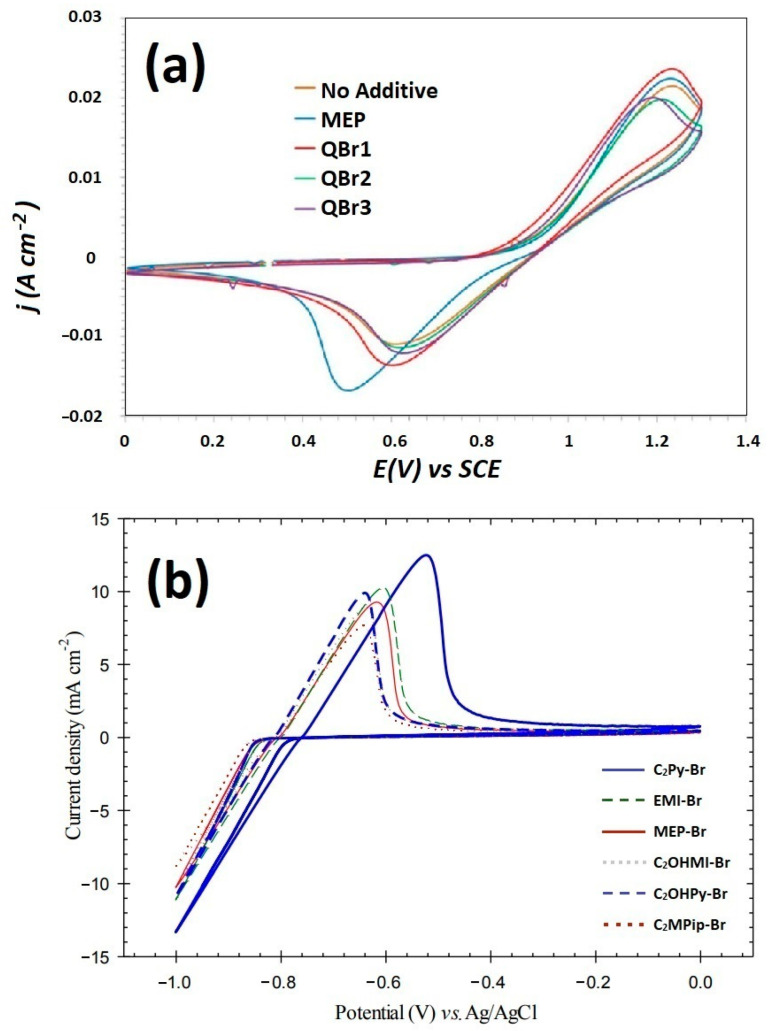
(**a**) CV at a GC electrode in 50 mM ZnBr_2_, 0.5 M KNO_3_ with 16.67 mM of MEP, CMMPy-Br (QBr1), CMMM-Br (QBr2) and CMMP-Br (QBr3) using a scan rate of 50 mV s^−1^ (T = 295 K) [46]; (**b**) CV measurement of Zn^2+^/Zn redox couple for C_2_Py-Br, EMI-Br, MEP-Br, C_2_OHMI-Br, C_2_OHPy-Br and C_2_MPip-Br BCAs [74]. ((**a**): reproduced with permission from Ref. [46] under the Creative Commons CC BY license. (**b**): reproduced with permission from Ref. [74]. © The Royal Society of Chemistry 2016).

On the one hand, D. Bryans et al. have shown cyclic voltammetry (Figure 4a) in the absence and presence of a 16.67 mM concentration of MEP-Br, CMMPy-Br (QBr_1_), CMMM-Br (QBr_2_) and CMMP-Br (QBr_3_). At the glassy carbon (GC) electrode, the Br^−^ oxidation reaction led to a diffusion-controlled current peak at ca. 1.22 V and the reverse Br_2_ reduction gave a peak at 0.62 V. The large peak separation suggests that the Br^−^/Br_2_ reaction at the GC electrode is electrochemically irreversible. The Br_2_ reduction peak in the presence of MEP-Br is higher than for all the other BCAs but also takes place at a more negative potential. Even though QBr_1_, QBr_2_ and QBr_3_ all generate very similar cyclic voltammograms in terms of the size and position of the oxidation and reduction peaks, visual control of the GC electrode after CV with these BCAs shows that a surface coating was also present, despite their reductive peaks maintaining a diffusion-controlled profile [46].

Other BCA-supported electrolytes are compared (see Figure 4b) with a Zn^2+^/Zn redox couple curve, and the results have been related with the nucleation overpotential (NOP) of the electrodeposited zinc. In this case, C_2_Py-Br, EMI-Br, MEP-Br, C_2_OHMI-Br, C_2_OHPy-Br and C_2_MPip-Br are the compared BCAs. According to G. P. Rajarathnam et al., the highest magnitude of the current densities obtained during zinc electrodeposition/stripping are for C_2_Py-Br, while the lowest values are for C_2_MPip-Br. This is because the relationship between a better zinc half-cell electrochemical performance and the narrowing of the nucleation overpotential makes C_2_Py-Br the lowest NOP producer. In general, a better zinc half-cell CV performance is observed in all the BCAs containing delocalized cationic charge (EMI-Br, C_2_Py-Br and C_2_OHPy-Br), compared with BCAs with a localized charge (MEP-Br and C_2_MPip-Br) [74].

These results are also in line with those obtained by Y. Lee et al. [33], who tested other BCA-supported (MEP-Br, MEM-Br and DMEI-Br) electrolytes using CV measurements, demonstrating that the electrochemical performance of any of these BCAs was better compared with those obtained with cells that did not use a BCA. This is because they contribute to raising the reaction kinetics and reversibility of the Zn^2+^/Zn redox species in working electrolytes [42,92,93]. In this case, among the three BCAs used, DMEI-Br compared with MEM-Br and MEP-Br showed the best peak current ratio of IPA/IPC = 0.98. This excellent effect of the DMEI-Br was attributed to the contribution of the two nitrogen atoms in the aromatic resonance ring, thus increasing the number of active sites and improving the hydrophilic property of the plated zinc surface for the reduction and oxidation reaction of Zn^2+^/Zn [94,95].

**Figure 5:**
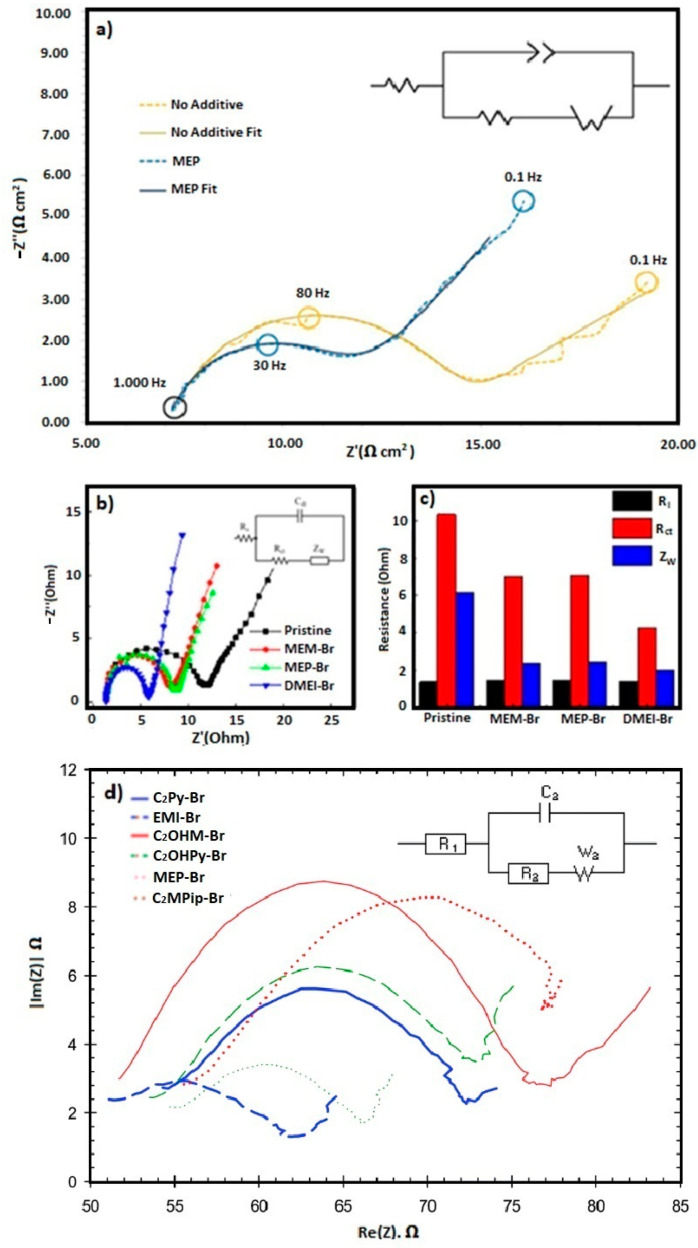
(**a**) EIS data obtained at E ½ in the solution containing 50 mM ZnBr_2_ and 0.5 M KNO_3_ without BCA (yellow) and with 16.67 mM MEP-Br (blue) at a GC electrode (T = 295 K) and the equivalent circuits for fitting the data [46]; (**b**) EIS comparisons of Nyquist plots for ZBFB static unit cells employing ZnBr_2_ electrolyte without (pristine) or with BCA (MEP-Br, MEM-Br and DMEI-Br) and (**c**) Rs, Rct and Zw for these cells [33]; (**d**) EIS comparisons of Nyquist plots of C_2_Py-Br, EMI-Br, MEP-Br, C_2_OHMI-Br, C_2_OHPy-Br and C_2_Mpip-Br [74]. ((**a**): reproduced with permission from Ref. [46] under the Creative Commons CC BY license. (**b**,**c**): reproduced with permission from Ref. [33]. © 2023 Elsevier B.V. All rights reserved. (**d**): reproduced with permission from Ref. [74]. © The Royal Society of Chemistry 2016).

The electrode kinetics of each system are investigated using EIS. D. Bryans et al. have compared some BCA-supported electrolytes, as seen in Figure 5a. The EIS spectra were carried out in electrolytes with and without MEP-Br at the half-wave potential, E_1/2_. In the presence of MEP-Br, the size of the “semi-circle” is smaller. The data are obtained by fitting an equivalent circuit including resistance (Rs) in series with a parallel RCT –W/CCPE circuit, correlated with the uncompensated solution resistance Rs between the RE and WE, the charge transfer resistance RCT, a constant phase element CPE and a Warburg diffusional impedance, W. This shows that the double-layer capacitance is five times larger for MEP-Br than that observed in solutions without a BCA or in solutions containing CMMPy-Br, CMMM-Br and CMMP-Br. This indicates that the formed MEP-Br layer on the electrode surface, once charged with the Br_2,_ is homogeneous to the one holding the Br_x_^−^ electroactive species at the electrode surface, but that the ones formed with CMMPy-Br, CMMM-Br and CMMP-Br are more weakly bound so that the reduction of the Br_x_^−^ species is controlled by diffusion back to the electrode surface [46].

EIS experiments have been carried out to evaluate and compare the electrolyte resistances and the charge transfer resistance according to the use of MEP-Br, MEM-Br and DMEI-Br as a BCA (0.6 M BCA in 2.0 M ZnBr_2_; see Figure 5b,c), where the frequency is changed from 0.001 Hz to 600 kHz at 10 mV of amplitude. Y. Lee et al. [33] have studied the solution resistance (Rs), the charge transfer resistance (Rct) and the Warburg diffusion resistance (Zw) as observed in a Nyquist diagram of the data from the measured impedance. From these results, the three BCA-supported electrolytes show an Rs like the pristine one (1.34 Ω) although a BCA is added. DMEI-Br exhibits the lowest Rct and Zw. This implies that the DMEI-Br has the most enhanced reaction kinetics and electron transfer of the Zn^2+^/Zn and Br_2_/Br^−^ redox couples, so promising a significant improvement in the coulombic efficiency of a ZBFB. Consequently, it is demonstrated that DMEI-Br (compared with MEP-Br and MEM-Br) facilitates faster kinetics and better electron charge transport in the anolyte and catholyte electrolytes, associated with homogeneous zinc-plating and polybromide phase dispersion [42,92,93].

G. P. Rajarathnam et al. have carried out EIS measurement to compare the resistance and charge transfer of electrolytes containing the following BCAs: C_2_Py-Br, EMI-Br, MEP-Br, C_2_OHMI-Br, C_2_OHPy-Br and C_2_MPip-Br (see Figure 5d). In this case, the electrolyte solution resistances are between 53 and 58 Ω, with C_2_MPip-Br having the highest one. A higher Rs suggests lower mobility of ions through the electrolyte under polarization conditions, which in turn can be attributed to physical properties of the electrolyte like lower ionic conductivity. This suggestion is supported by the 9% lower ionic conductivity showed for the electrolyte containing C_2_Mpip-Br compared to the electrolyte using MEP-Br. The better-performing electrolytes (EMI-Br, C_2_Py-Br and C_2_OHPy-Br) showed conductivities 5–7% higher than MEP-Br, although they also produced a higher Rs. A tendency is observed between the charge transfer resistances (Rct) and the structures of the BCAs. The electrolytes containing the two BCAs with hydroxyl functional groups (C_2_OHMI-Br and C_2_OHPy-Br) produced the lowest Rct values. The use of a non-hydroxyl kind of these BCAs concluded in an increase in these values (EMI-Br and C_2_Py-Br), while electrolytes containing MEP-Br and C_2_Mpip-Br produced the highest Rct. This suggest the strong dependence of the zinc-side charge transfer resistance on other factors (pH, conductivity or other factors related to the chemical structure of the BCAs) that are unable to be measured directly. In addition, the EIS results also showed that the use of non-commercial BCAs decreases the resistance associated with the Warburg diffusion limitation (Zw) in the zinc half-cell. This observation suggests that BCAs interact with the ions in the solution and strongly influence ion action (including mobility) [74].

### 4.4. Cell Performance in ZBFBs

For the evaluation of the binding strength of BCAs to bromine molecules, long-term cycling tests are carried out using an operative ZBFB. In addition, the coulombic, voltaic and energy efficiencies of all the studied BCAs are obtained and compared between them. Energy efficiency is an important battery performance parameter because it indicates the energy losses incurred during charging and discharging. A higher voltaic efficiency suggests that a battery suffers fewer losses from fast charging and discharging and allows a higher power draw. A higher coulombic efficiency shows that a battery suffers lower self-discharge. The voltaic efficiency depends mostly on the bromine supply to the electrode during discharge, as shown above [34].

**Figure 6:**
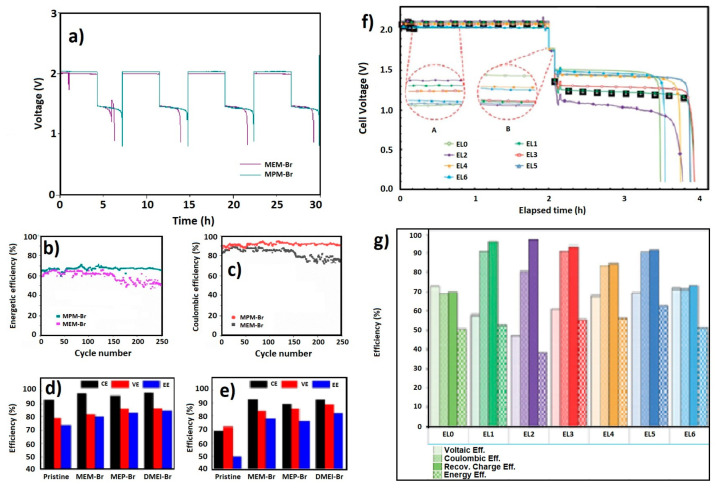
(**a**) Charge–discharge profile, (**b**) energy efficiency and (**c**) coulombic efficiency of MEM-Br- and MPM-Br-supported electrolytes on ZBFB at 1 M, 2 M ZnBr_2_ and 20 mA/cm^2^ current density [21]; (**d**) efficiency comparison (298 K) and (**e**) efficiency comparison (333 K) of MEM-Br, MEP-Br, DMEI-Br and pristine electrolytes [33]; (**f**) comparison of final cycle voltage with different BCAs and (**g**) cycle performance of EL0 (without BCA), EL1 (MEP-Br), EL2 (C_2_MPip-Br), EL3 (C_2_Py-Br), EL4 (C_2_OHPy-Br), EL5 (EMI-Br) and EL6 (C_2_OHMI-Br) [34]. ((**a**–**c**): reproduced with permission from Ref. [21] under the Creative Commons CC BY license; (**d**,**e**): reproduced with permission from Ref. [33]. © 2023 Elsevier B.V. All rights reserved; (**f**,**g**): reproduced with permission from Ref. [34]. © The Royal Society of Chemistry 2016).

U. Jiménez-Blasco et al. have cycled and compared the BCA analogs MEM-Br and MPM-Br to study the effect of the aliphatic chain growth. In Figure 6a–c, respectively, the charge–discharge profile, energy efficiency and coulombic efficiency during 250 cycles of both BCAs can be seen. In this case, it is noted that MPM-Br has a lower resistance compared with MEM-Br because the voltage is lower during charge and higher during discharge. Consequently, both the coulombic and energy efficiency increase for the MPM-Br. With certainty, it can be said that with a longer aliphatic chain, more free bromine can be complexed with the BCA [21].

MEM-Br, MEP-Br and DMEI-Br have been compared by Y.Lee et al., using them as BCAs in ZBFBs. Figure 6d,e show the coulombic (CE), voltaic (VE) and energy (EE) efficiencies for cells built using each one of these three BCAs and in the absence of BCA, at 298 K and 333 K, respectively. To carry out this performance, 6 cm^2^ active area electrodes are used for 200 cycles, where the charge conditions are 20 mAcm^2^ and 1 h/cycle. Additionally, comparisons are made with three 2.0 M ZnBr_2_ electrolyte solutions of an initial pH = 1, using 0.6 M of each BCA. A full cell experiment at 333 K is implemented because, during charge, the bromine loss (Br_2_ evaporation at high temperature) occurs at the positive electrolyte. With long-term cycling, this is a major contributor to the decreased columbic efficiency of the flow cell. Because of this, it is necessary to verify the bromine-capturing capacity and bromine-binding stability of BCAs at those temperatures. The addition of a BCA reagent improved the CE, VE and EE values compared to the pristine control. This means that BCA-supported electrolytes had improved electrochemical characteristics in terms of the kinetics and the reversibility of Zn^2+^/Zn and Br_2_/Br^−^ redox couples. In general, in Figure 6e, the best efficiency (in most cases) is seen when DMEI-Br is used as a BCA, compared with the other BCAs tested in this work [33].

M. Schneider et al. have used C_2_Py-Br, EMI-Br, MEP-Br, C_2_OHMI-Br, C_2_OHPy-Br and C_2_MPip-Br as BCAs in ZBFBs to compare between them. The results obtained indicate that the studied BCAs have an influence on the efficiencies in different ways. Figure 6f,g compare the cell voltage progression for the fourth cycle of each electrolyte. The differences in cell polarization during the discharge phase are much larger than during the charge phase. EL0 (without a BCA) has the lowest charge voltage and the highest discharge voltage. Therefore, the addition of a BCA increases the cell polarization. The cell voltages remained stable during the charging process. There are 100 mV differences in the charging voltages, with the lowest charging voltages for EL0, EMI-Br and C_2_OHMI-Br. The voltages for C_2_Py-Br and C_2_OHPy-Br are similar and between the two extremes. The electrode polarization for EL0 during the charge process can be attributed to the ohmic resistances of the electrolyte and the electrode. EL0 performed best in terms of VE, followed by C_2_OHMI-Br, EMI-Br and C_2_OHPy-Br. The VE of C_2_Py-Br and MEP-Br is lower, and C_2_MPip-Br has the lowest VE of all the electrolytes. This is generally reversed for CE, as MEP-Br, C_2_Py-Br and EMI-Br have the highest and EL0 and C_2_OHMI-Br the lowest CE. C_2_OHPy-Br and C_2_MPip-Br CE are between these extremes. However, for C_2_MPip-Br, low bromine release is the limiting factor, while for all other electrolytes, it is zinc availability, as influenced by the recoverable charge share. Recoverable charge (RC) is a good indicator of the complexation efficiency because it is correlated with zinc availability. Bromine-induced self-discharge is considered the dominant side reaction. As expected, the recoverable charge share for EL0 is the lowest. C_2_MPip-Br has the highest RC share and CE, compared to all other electrolytes. In terms of EE, C_2_MPip-Br has the poorest performance, because of the insufficient bromine release during discharge. EMI-Br has the highest EE since it had good performance according to both parameters. C_2_Py-Br and C_2_OHPy-Br also performed better than MEP-Br. However, the higher self-discharge of C_2_OHPy-Br made it less suitable for energy storage compared to C_2_Py-Br. The addition of a BCA has reduced self-discharge, but has also caused cell polarization (C_2_MPip-Br and MEP-Br differed from the other BCAs in their localized cationic charge). They caused the strongest cell polarization, but had a better complexation efficiency than the other electrolytes. C_2_Py-Br and EMI-Br with a delocalized cationic charge demonstrated lower cell polarization, but also reduced complexation efficiency. The presence of an OH group increases these effects, as evident with C_2_OHPy-Br and C_2_OHMI-Br [34].

Considering the aforementioned factors, Table 5 shows a summary of the efficiency parameters obtained for all the studied BCAs used in the electrolyte on the ZBFB performance. As can be seen, although commercial BCAs such as MEM-Br or MEP-Br are those that report the best efficiency results, some of the studied BCAs present promising results. One of the BCAs with the best efficiency values is DMEI-Br, with which better results have been obtained compared to commercial ones, obtaining a coulombic efficiency of 95%, voltaic efficiency of 85% and energy efficiency of 80%. Interesting results have been obtained with other BCAs such as MPM-Br, EMI-Br or C_2_Py-Br, although the efficiency values were lower than those obtained with the commercial ones.

## 5. Overview and Future Perspectives

In general, ZBFBs can be considered one of the most promising technologies for stationary energy storage. Even though advancements have been realized, there are still some barriers to overcome to further accelerate the commercialization process, according to Y. Yin et al. [96].

On the one hand, it is necessary to optimize the fabrication of the cell design. From a practical point of view, it is difficult to meet all the requirements of the market, but it is known that a competitive ZBFB must meet the following requirements: low cost, high security, long life cycle, high energy density, simplicity, easy maintenance and excellent environmental suitability. It is necessary to look for innovative cell designs to compete with other energy storage technologies that solve the dendritic growth of zinc, the limitation in the capacity of the battery dictated by the area of the anode or the low energy density.

On the other hand, in terms of the electrolyte additives, there are new complexing agents and other additives that can affect the cell performance of ZBFBs. These additives can work in three ways, on the one hand, altering the complexing state of the polybromide, which enhances the bromine complexation capacity, increasing the ZBFB efficiency. On the other hand, additives can affect the deposited zinc morphology, optimizing the morphology of the electrochemical zinc deposition layer on the anode. Therefore, the reaction reversibility, area capacity and dendrite-free morphology will improve. Finally, additives can change some of the properties of the electrolyte such as the ion conductivity, kinetics and high and low temperature tolerance.

Thirdly, they could improve the anode with an increase in the effective area of the electrode and homogeneous (non-dendritic) deposition of zinc. Conductive anodes play the main role in ZBFBs in terms of the zinc deposition during charging. However, (non-uniform) dendritic deposition affects the cyclic stability and coulombic efficiency of the battery, with an increased impact the greater the effective area of the anode. To improve the energy density and decrease the cost of the ZBFB system, the following strategies should be considered: the functionalized surface of the anode should be further optimized to induce uniform zinc deposition; it is worth designing an advanced anode structure to provide a sufficient junction area and space to store zinc and complementary, innovative carbon-free anode materials could be a good alternative to alleviate zinc deposition problems.

To increase the current density of ZBFBs, it is necessary to improve the Br_2_/Br^−^ electrochemical reaction rate by optimizing the cathode. The material for an optimal activated cathode should have high selectivity to catalyze the Br_2_/Br reaction, high efficiency, high conductivity and high durability.

Finally, the design and synthesis of membranes with advanced materials has great importance because excellent mechanical and chemical stability is a prerequisite to resist zinc dendritic growth and bromine corrosion. In addition to this, the membrane must possess both high ionic conductivity and good resistance to polybromide crossover, as mentioned above.

To summarize, zinc–bromine redox flow batteries must use a bromine complexing agent as an additive for bromine stability. Nevertheless, the chemical and structural characteristics of the BCA considerably affect the performances of ZBFBs in different ways. Some BCAs have been studied in this review, with the intention of choosing the optimized one to improve ZBFB performance. To make this choice, some characteristics of BCAs have been compared, such as the kinetics of the reaction and reversibility of the Zn^2+^/Zn and Br_2_/Br^−^ redox couples, electrolyte conductivity and charge transfer resistance, deposited zinc morphology and zinc plating/stripping reversibility, bromine complexation capacity and complex stability, among others.

All the proposed BCAs have been compared with commercial ones (MEM-Br and MEP-Br) to check whether there is an improvement in ZBFB performance. In this case, these are proposed BCAs: MPM-Br, DMEI-Br, CMMPy-Br, CMMM-Br, CMMP-Br, C_2_Py-Br, EMI-Br, C_2_OHMI-Br, C_2_OHPy-Br and C_2_MPip-Br. Although it was impossible to compare all of them, in general, it can be said that all the studied BCAs show interesting characteristics in replacing the commercial ones. Specifically, MPM-Br, EMI-Br and DMEI-Br demonstrate significant advantages compared with the other BCAs.

In conclusion, it can be said that, although the current research has found promising results, there is still a long way to go in terms of better ZBFB performance. Even so, increasingly competitive batteries are being obtained on the storage systems market due to the advantages that this technology presents, such as the low cost of the raw materials used, scalability and specific design capacity, which can be adjusted to the needs of each user. For this reason, the commercialization of ZBFBs continues to grow every year.

## Figures and Tables

**Table 2:** Summary of different syntheses of MPM-Br.

Temp. (°C)	Solvent (mL/g reac.)	Adduct Excess (%)	N_2_	Reflux	Crystallization (h)	Yeld (%)
70	1.41	14	Yes	Yes	12	68
70	1.11	60	Yes	Yes	12	85
70	0	60	Yes	Yes	12	19
50	1.11 *	60	Yes	Yes	12	10
70	0.55	60	Yes	Yes	2	90
25	0.59	50	No	No	120	89

* Acetone was used as the solvent instead of ACN.

**Table 3:** Summary of ionic liquids studied to use as BCAs in ZBFBs.

BCA	Molecule	Precursor	Synthesis Conditions				Dried under Vacuum	Ref.
			T (°C)	Time (h)	Refl.	Atm		
MEM-Br	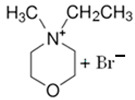	-N-methylmorpholinium -bromoethane -acetonitrile	65	5	Yes	N_2_	Yes	[64,85,86]
MPM-Br	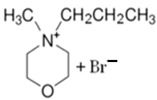	-4-methylmorpholinium -1-bromopropane -acetonitrile	Room T	120	No	No	Yes	[21]
CMMM-Br	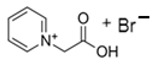	-4-methylmorpholinium -3-bromopropanoic acid	Room T	4	No	No	Yes	[46,87]
MEP-Br	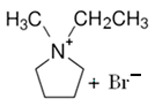	-N-methypyrrolidinium -bromoethane -acetonitrile	Room T	24	No	No	Yes	[60,67]
CMMP-Br	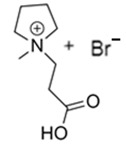	-N-metilpyrrolidinium -3-bromopropanoic acid	Room T	4	No	No	Yes	[46,87]
EMI-Br	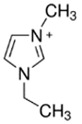	-3-methilimidazolium -bromoethane	Room T	24	Yes	No	Yes	[34,75]
DMEI-Br	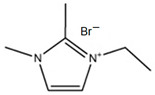	-1,2-dimethylimidazolium -bromoethane	45	12	Yes	No	No	[33,76,77]
C_2_OHMI-Br	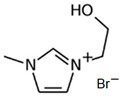	-3-methilimidazolium -bromoethanol	Room T	24	Yes	No	Yes	[34,75]
C_2_MPip-Br	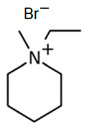	-N-methylpiperidinium -bromoethane	75–80	48	Yes	N_2_	Yes	[74,75,80]
C_2_Py-Br	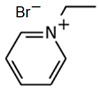	-pyridine -bromoethane	Room T	24	Yes	No	Yes	[34,75]
C_2_OHPy-Br	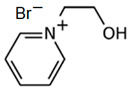	-pyridine -bromoethanol	Room T	24	Yes	No	Yes	[34,75]
CMMPy-Br	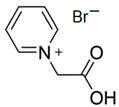	-pyridine -2-bromoacetic acid	-	4	No	No	Yes	[46]

**Table 4:** Raman intensities for polybromide species.

Polybromide Species	Raman Shift (cm^−1^)
Tribromide (Br_3_^−^)	163–198
Pentabromide (Br_5_^−^)	231–380
Free bromine (Br_2_)	310

**Table 5:** Average value of CE, VE and EE obtained for ZBFB.

BCA	Structure	Coulombic Efficiency (%)	Voltaic Efficiency (%)	Energy Efficiency (%)
MEM-Br	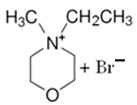	90.7	80	78.1
MEP-Br	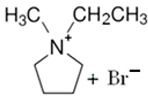	91.1	58.1	52.8
MPM-Br	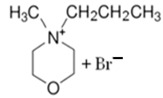	90	-	70
DMEI-Br	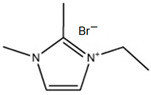	95	85	80
C_2_OHPy-Br	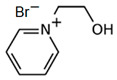	83.4	67.8	56.6
C_2_OHMI-Br	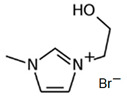	71.4	71.7	51.3
C_2_MPip-Br	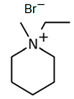	80.3	47.4	38.4
C_2_Py-Br	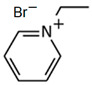	91.1	61.1	55.8
EMI-Br	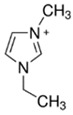	90.6	69.5	63

## Data Availability

Not applicable.
